# Supplementary figures and images for: Bio-On-Magnetic-Beads (BOMB): Open platform for high-throughput nucleic acid extraction and manipulation
Source: PLoS Biol. 2019 Jan 10;17(1):e3000107. doi: 10.1371/journal.pbio.3000107 (PMC6343928; doi:10.1371/journal.pbio.3000107)

## A Magnetic core particles

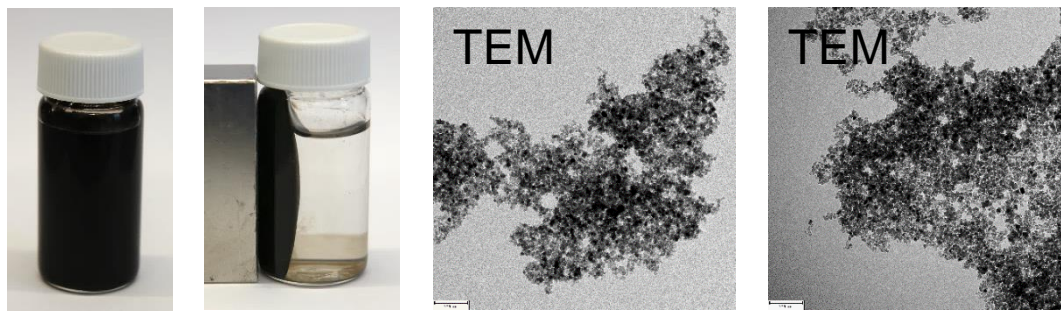

## B Silica-coated beads

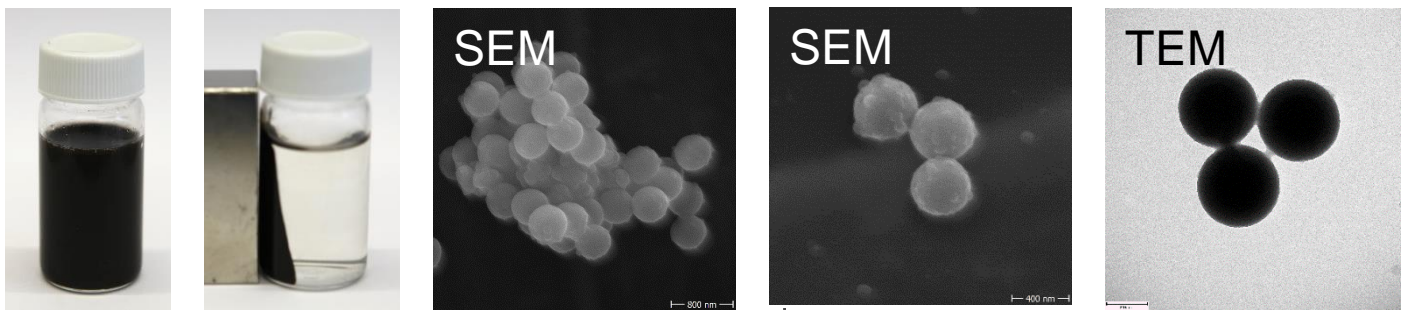

## C Carboxyl-coated beads

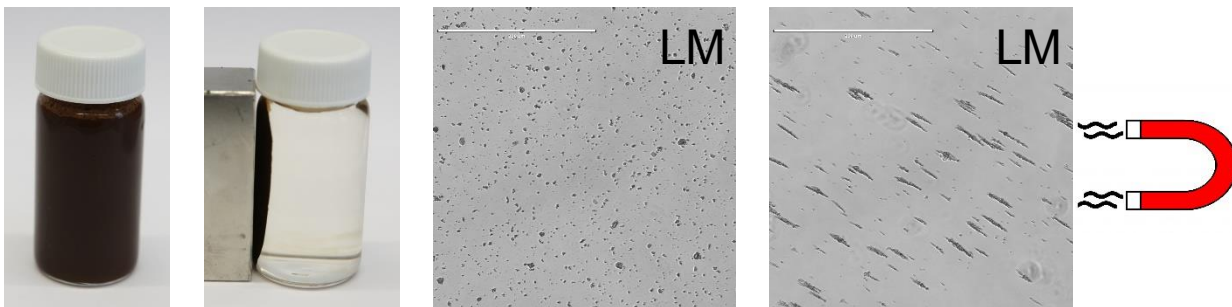

Supplement: S1 Fig — (A) Magnetic core particles (see S1 Appendix, BOMB protocol 1.1) in TEM. (B) Silica-coated magnetic beads (see S1 Appendix, BOMB protocol 2.1) in SEM and TEM. (C) Carboxyl-coated magnetic beads (see S1 Appendix, BOMB protocol 3.1) in LM, with and without an applied magnet. BOMB, Bio-On-Magnetic-Beads; LM, light microscopy; SEM, scanning electron microscopy; TEM, transmission electron microscopy. (PDF) [file pbio.3000107.s001.pdf]

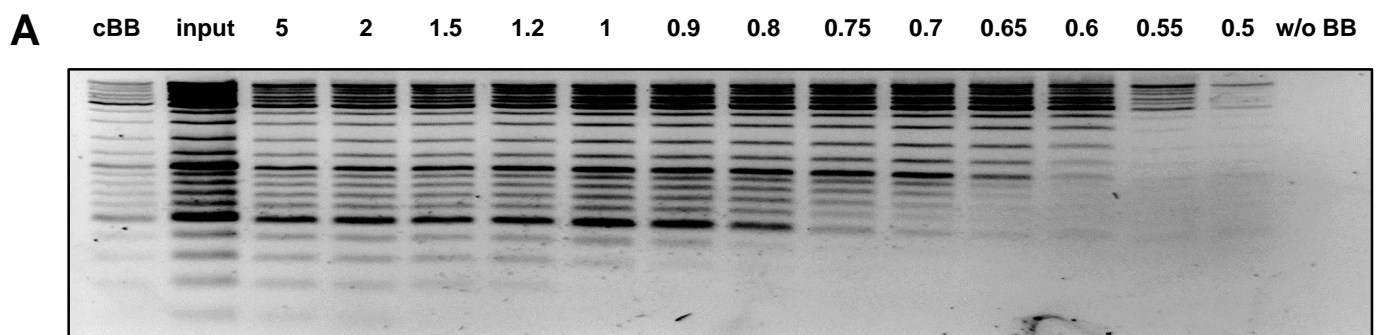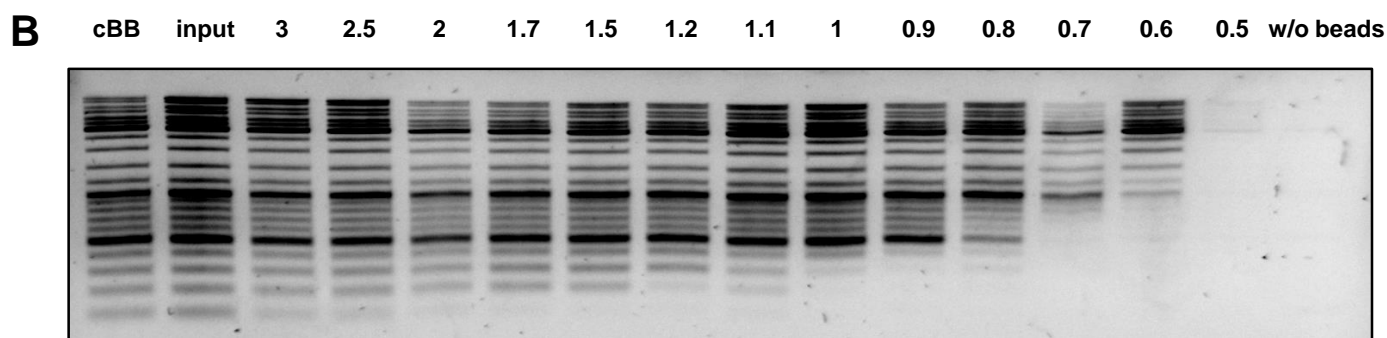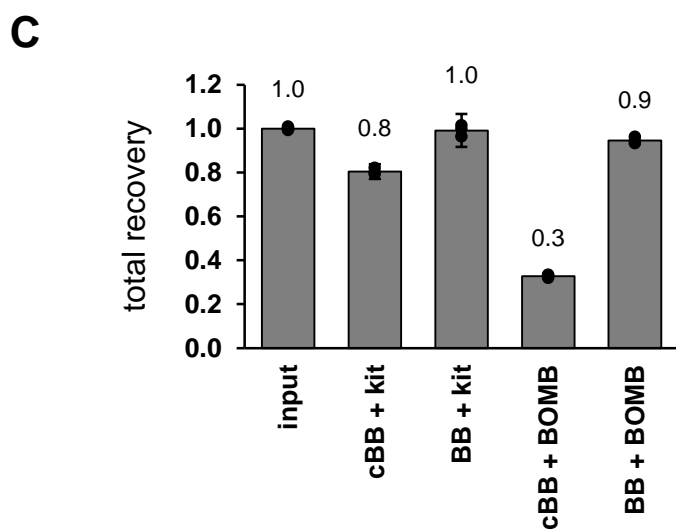

Supplement: S2 Fig — (A) Size exclusion of GeneRuler DNA Ladder Mix (Thermo) using silica-coated magnetic beads (see S1 Appendix, BOMB protocol 4.1). Different volumes of BB compared to sample volume were used to achieve size exclusion; as a comparison, 2 volumes of cBB were used or no binding buffer was included (w/o BB) (B) Size exclusion of GeneRuler DNA Ladder Mix using carboxyl-coated magnetic beads (see S1 Appendix, BOMB protocol 4.2). Different volumes of BB compared to sample volume were used to achieve size exclusion; as a comparison, 3 volumes of cBB were used or no beads were included (w/o beads). (C) Total recovery of approximately 6 μg plasmid DNA (input) using either a commercial kit that includes silica-packed columns (kit) or the S1 Appendix, BOMB protocol 4.1 for clean-up with silica-coated beads (BOMB). For binding, either cBB or the BB described in the BOMB protocol above was used. Error bars represent standard deviation, n = 3. Underlying data for S2 Fig can be found in S2 Data. BB, binding buffer; BOMB, Bio-On-Magnetic-Beads; cBB, commercial binding buffer. (PDF) [file pbio.3000107.s002.pdf]

**A**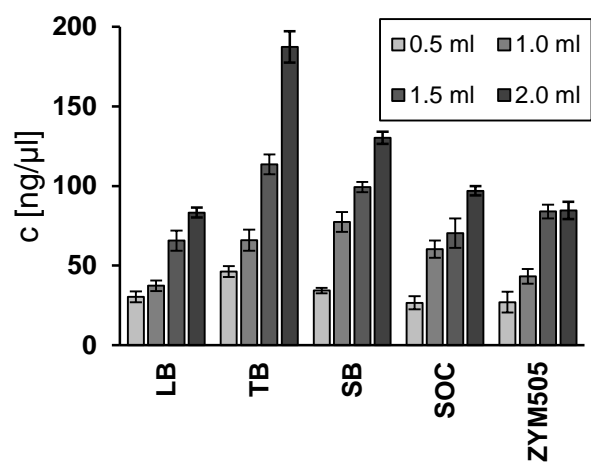**B**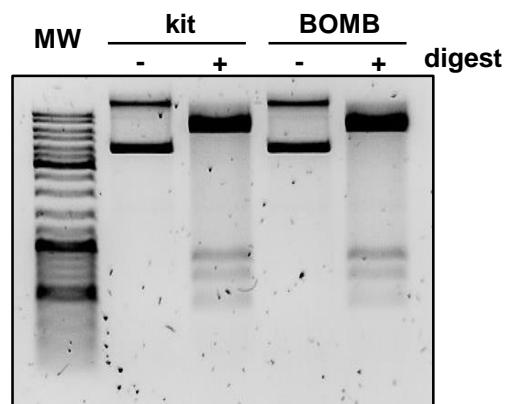**C**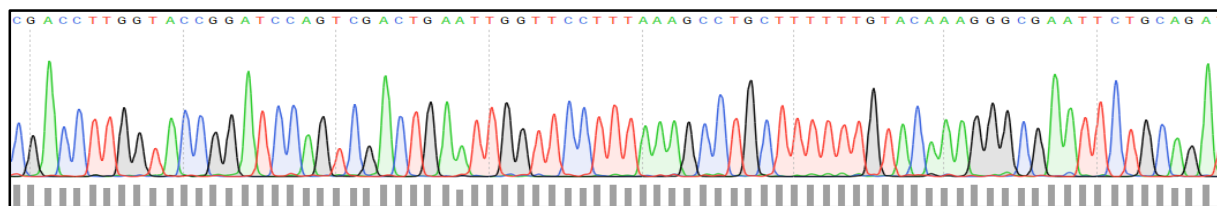

Supplement: S3 Fig — (A) Optimisation of reaction volume and media. One colony was picked and used to inoculate a 5 ml preculture containing LB media and ampicillin. The culture was incubated at 37 °C and 250 rpm until an OD of 0.6 was reached; 5 μl of the preculture was used to inoculate different volumes (0.5 ml, 1.0 ml, 1.5 ml, and 2.0 ml) of a variety of growth media (LB, TB, SB, SOC, and ZYM505, including the respective antibiotic) in a 2.2 ml 96-well culture plate (Sarstedt). The plate was sealed with the lid of a 6-well cell culture plate, so a decent exchange of air was possible, and incubated at 37 °C and 250 rpm for 22 hours. Plasmid DNA was then isolated with the S1 Appendix, BOMB protocol 5.1, and the resulting concentration (c [ng/μl]) was determined. Error bars represent the standard deviation, n = 3. (B) Comparison of commercially purified plasmid DNA (kit) and BOMB extracted DNA (BOMB) with (+) and without (−) restriction enzyme digestion. MW: GeneRuler DNA Ladder Mix (Thermo Scientific). (C) Exemplary sequencing trace of a BOMB extracted plasmid via Sanger sequencing. A sequencing read length of at least 800–1,000 bp is typically observed. Underlying data for S3 Fig can be found in S3 Data. BOMB, Bio-On-Magnetic-Beads; LB, Luria-Bertani Broth; TB, Terrific Broth;SB, Super Broth; SOC, Super Optimal Broth. (PDF) [file pbio.3000107.s003.pdf]

MW

Clover leaf gDNA

MW

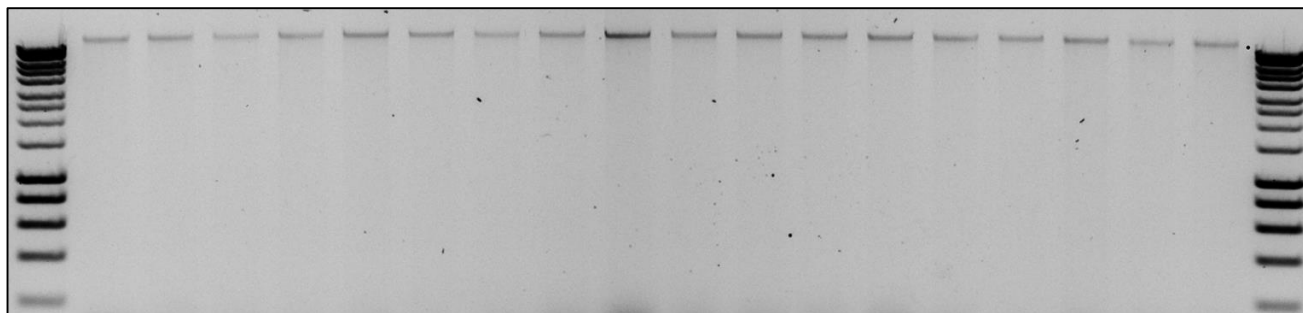

Supplement: S5 Fig — Genomic DNA was isolated from individual leaves (approximately 5 mg of tissue) using S1 Appendix, BOMB protocol 6.4 (high-throughput). A subset of DNA samples (18) from 96 extractions, represented in Fig 2O, were run on an agarose gel. MW: Hyperladder I (Bioline). BOMB, Bio-On-Magnetic-Beads. (PDF) [file pbio.3000107.s005.pdf]

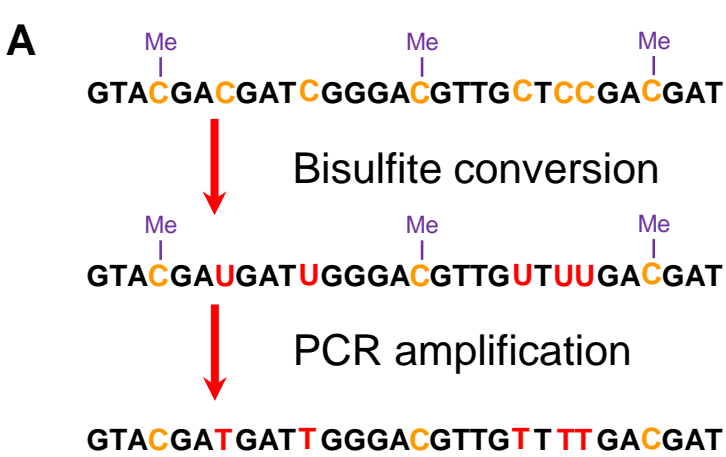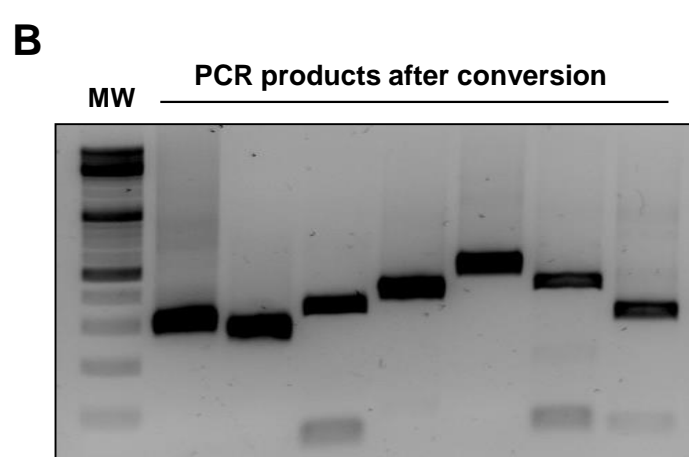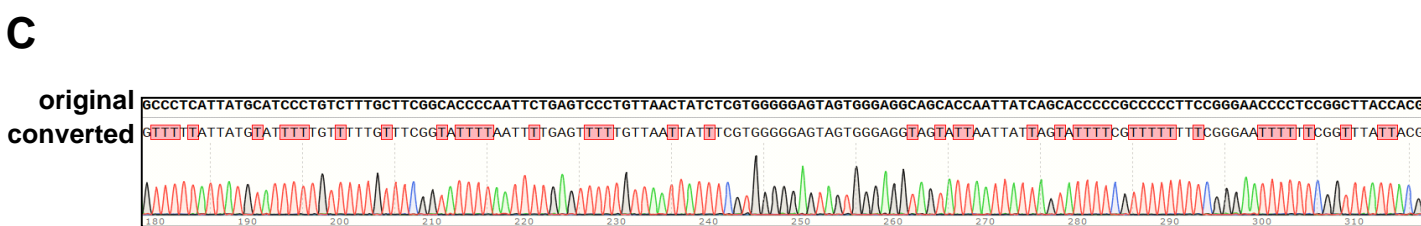

Supplement: S6 Fig — (A) Scheme of DNA methylation analysis using bisulfite conversion. (B) Agarose gel after PCR amplification of bisulfite-converted human DNA. Multiple primer pairs with expected product sizes between 221 and 435 bp were tested successfully. MW: GeneRuler DNA Ladder Mix (Thermo Scientific). (C) Sequencing trace of a PCR-amplified bisulfite converted sample, aligned with the original, unconverted sequence. All non-CG cytosines were successfully converted. Conversion rate is approximately 99% as measured by Sanger sequencing after PCR amplification. BOMB, Bio-On-Magnetic-Beads; CG, cytosine-guanine dinucleotide. (PDF) [file pbio.3000107.s006.pdf]
