# Supplementary material for: Bio-On-Magnetic-Beads (BOMB): Open platform for high-throughput nucleic acid extraction and manipulation
Source: PLoS Biol. 2019 Jan 10;17(1):e3000107. doi: 10.1371/journal.pbio.3000107 (PMC6343928; doi:10.1371/journal.pbio.3000107)
Supplement: S4 Fig — Genomic DNA was isolated from the indicated tissues of a 12-hour deceased rabbit, using S1 Appendix, BOMB protocol 6.3 and (A) Speed Beads or (B) BOMB silica beads. A comparison to (C) phenol-chloroform–based extraction is also shown. MW in all panels represents Hyperladder I (Bioline). Inevitably, some tissues (like bone marrow) produce far greater (D) yields per mg of input material, compared to other tissues. However, the bead-based methods generally outperform phenol-chloroform extractions in our hands. Note: rabbit tissues were not preserved immediately after animal death, hence why tissues like spleen have experienced some DNA degradation. Underlying data for S4 Fig can be found in S4 Data. BOMB, Bio-On-Magnetic-Beads. (PDF) [file pbio.3000107.s004.pdf]

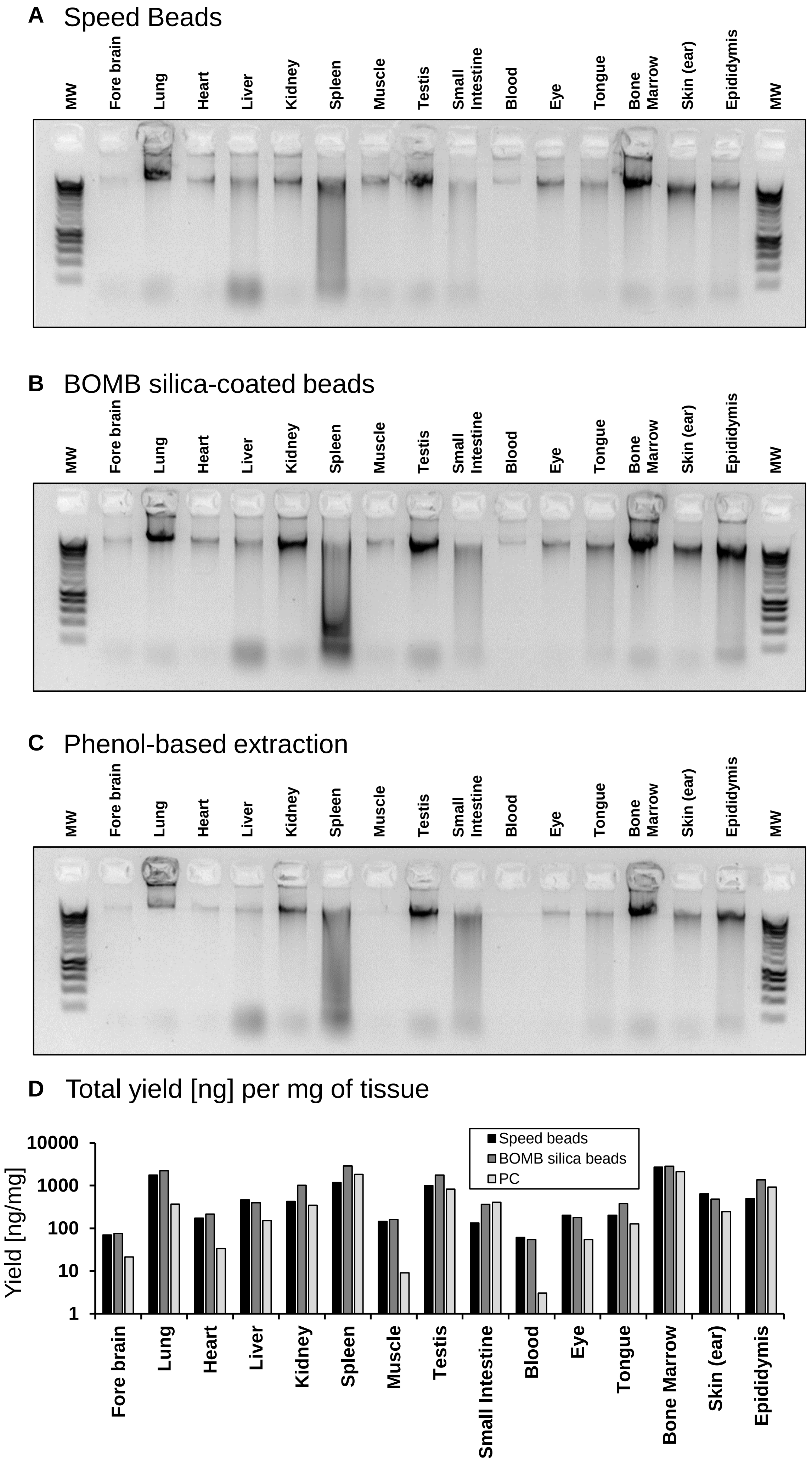
A Speed Beads
MW
Fore brain
Lung
Heart
Liver
Kidney
Spleen
Muscle
Testis
Small Intestine
Blood
Eye
Tongue
Bone Marrow
Skin (ear)
Epididymis
MW
B BOMB silica-coated beads
MW
Fore brain
Lung
Heart
Liver
Kidney
Spleen
Muscle
Testis
Small Intestine
Blood
Eye
Tongue
Bone Marrow
Skin (ear)
Epididymis
MW
C Phenol-based extraction
MW
Fore brain
Lung
Heart
Liver
Kidney
Spleen
Muscle
Testis
Small Intestine
Blood
Eye
Tongue
Bone Marrow
Skin (ear)
Epididymis
MW
D Total yield [ng] per mg of tissue
Speed beads
BOMB silica beads
PC
Yield [ng/mg]
10000
1000
100
10
1
Fore brain
Lung
Heart
Liver
Kidney
Spleen
Muscle
Testis
Small Intestine
Blood
Eye
Tongue
Bone Marrow
Skin (ear)
Epididymis
