# Supplementary material for: Bio-On-Magnetic-Beads (BOMB): Open platform for high-throughput nucleic acid extraction and manipulation
Source: PLoS Biol. 2019 Jan 10;17(1):e3000107. doi: 10.1371/journal.pbio.3000107 (PMC6343928; doi:10.1371/journal.pbio.3000107)
Supplement: S1 Appendix — (DOCX) [file pbio.3000107.s011.docx]

### BOMB protocols and files

### Magnetic beads

BOMB protocol 1.1 - [Magnetic nano particles (MNP) synthesis](https://bomb.bio/wp-content/uploads/2018/11/1.1_BOMB_MNP_synthesis_V1.2.pdf)

BOMB protocol 2.1 - [Silica coating of MNPs](https://bomb.bio/wp-content/uploads/2018/11/2.1_BOMB_coating_of_ferrite_MNPs_with_silica_V1.2.pdf)

BOMB protocol 3.1 - [Carboxyl coating of MNPs](https://bomb.bio/wp-content/uploads/2018/09/3.1_BOMB_MNP_coating_carboxyl_groups_V1.0.pdf)

### Nucleic acid extraction

BOMB protocol 4.1 - [Clean up silica-coated MNPs](https://bomb.bio/wp-content/uploads/2018/09/4.1_BOMB_clean_up_size_exclusion_silica_V1.0.pdf)

BOMB protocol 4.2 - [Clean up carboxyl-coated MNPs](https://bomb.bio/wp-content/uploads/2018/11/4.2_BOMB_clean-up_size_exclusion_carboxyl_V1.1.pdf)

BOMB protocol 4.3- [Agarose gel extraction](https://bomb.bio/wp-content/uploads/2018/11/4.3_BOMB_Agarose_extraction_V1.1.pdf)

BOMB protocol 5.1- [Plasmid DNA extraction](https://bomb.bio/wp-content/uploads/2018/09/5.1_BOMB_plasmid_DNA_extraction_V1.0.pdf)

BOMB protocol 5.2 - [Plasmid DNA extraction using MNP-pulldown](https://bomb.bio/wp-content/uploads/2018/10/5.2_BOMB_plasmid_DNA_extraction_using_MNP-pulldown_V1.0.pdf)

BOMB protocol 6.1 - [TNA extraction from mammalian cells using GITC](https://bomb.bio/wp-content/uploads/2018/09/6.1_BOMB_TNA_extraction_mammalian_GITC_v1.0.pdf)

BOMB protocol 6.2 - [TNA extraction from mammalian cells using TRI reagent](https://bomb.bio/wp-content/uploads/2018/09/6.2_BOMB_TNA_extraction_mammalian_TRI_v1.0.pdf)

BOMB protocol 6.3 - [TNA extraction from tissues](https://bomb.bio/wp-content/uploads/2018/11/6.3_BOMB_TNA_extraction_mammalian_tissue_GITC_V1.1.pdf)

BOMB protocol 6.4 - [TNA extraction from plants](https://bomb.bio/wp-content/uploads/2018/11/6.4_BOMB_TNA_extraction_plant_GITC_V1.1.pdf)

BOMB protocol 6.5 - [TNA extraction from yeast](https://bomb.bio/wp-content/uploads/2018/09/6.5_BOMB_TNA_extraction_yeast_GITC_V1.0.pdf)

BOMB protocol 6.6 - [TNA extraction from E.coli](https://bomb.bio/wp-content/uploads/2018/09/6.6_BOMB_TNA_extraction_Ecoli_TRI_V1.0.pdf)

BOMB protocol 6.7 - [TNA extraction from environmental samples](https://bomb.bio/wp-content/uploads/2018/11/6.7_BOMB_TNA_extraction_environmental_v1.1.pdf)

BOMB protocol 7.1 - [gDNA extraction from mammalian cells](https://bomb.bio/wp-content/uploads/2018/09/7.1_BOMB_genomic_DNA_extraction_v1.0.pdf)

BOMB protocol 8.1 - [RNA extraction from mammalian cells using TRI reagent](https://bomb.bio/wp-content/uploads/2018/09/8.1_BOMB_total_RNA_extraction_mammalian_TRI_V1.0.pdf)

BOMB protocol 8.2 - [RNA extraction from mammalian cells using GITC](https://bomb.bio/wp-content/uploads/2018/09/8.2_BOMB_total_RNA_extraction_mammalian_GITC_V1.0.pdf)

BOMB protocol 9.1 - [Bisulfite conversion](https://bomb.bio/wp-content/uploads/2018/09/9.1_BOMB_bisulfite_conversion_V1.0.pdf)

### Magnetic racks

BOMB protocol A.1 - [3D printed magnetic racks](https://bomb.bio/wp-content/uploads/2018/11/A.1_BOMB_magnetic_racks_V1.1.pdf)

BOMB protocol A.2 - [Laser-cut ring magnetic racks](https://bomb.bio/wp-content/uploads/2018/09/A.2_BOMB_ring_magnetic_racks_V1.0.pdf)

### Reagents

BOMB protocol B.1 - [TRI reagent](https://bomb.bio/wp-content/uploads/2018/09/B.1_BOMB_TRI_reagent_V1.0.pdf)

### BOMB rack files

BOMB file1 - [BOMB 6 button compact magnetic rack](https://bomb.bio/wp-content/uploads/2018/08/BOMBRack_6buttonScrew_8mm_compact.dxf)

BOMB file2 - [BOMB 6 button magnetic rack](https://bomb.bio/wp-content/uploads/2018/08/BOMBRack_6buttonScrew_8mm.dxf)

BOMB file3 - [BOMB microplate magnetic rack](https://bomb.bio/wp-content/uploads/2018/08/BOMB-microplate.stl)

BOMB file4 - [BOMB 8×1.5 magnetic rack](https://bomb.bio/wp-content/uploads/2018/08/A_BOMB-8×1.5-magnetic-rack.stl)
